# The Interplay Between Keratinocytes and Immune Cells in the Pathogenesis of Psoriasis

**DOI:** 10.3389/fimmu.2018.01549

**Published:** 2018-07-06

**Authors:** Cristina Albanesi, Stefania Madonna, Paolo Gisondi, Giampiero Girolomoni

**Affiliations:** ^1^Laboratory of Experimental Immunology, Istituto Dermopatico dell’Immacolata (IDI), IRCCS, Rome, Italy; ^2^Section of Dermatology, Department of Medicine, University of Verona, Verona, Italy

**Keywords:** psoriasis, keratinocytes, immune cells, skin inflammation, innate immunity, adaptive immunity

## Abstract

Psoriasis is a chronic inflammatory skin disease resulting from genetic, epigenetic, environmental, and lifestyle factors. To date, several immunopathogenic mechanisms of psoriasis have been elucidated, and, in the current model, the cross talk between autoreactive T cells and resident keratinocytes generates inflammatory and immune circuits responsible for the initiation, progression, and persistence of the disease. Several autoantigens derived from keratinocytes (i.e., LL37 cathelecidin/nucleic acid complexes, newly generated lipid antigens) have been identified, which may trigger initial activation of T cells, particularly IL-17-producing T cells, T helper (Th)1 and Th22 cells. Hence, lymphokines released in skin lesions are pivotal for keratinocyte activation and production of inflammatory molecules, which in turn lead to amplification of the local immune responses. Intrinsic genetic alterations of keratinocytes in the activation of signal transduction pathways dependent on T-cell-derived cytokines are also fundamental. The current review emphasizes the aberrant interplay of immune cells and skin-resident keratinocytes in establishing and sustaining inflammatory and immune responses in psoriasis.

## Introduction

**Figure 1:**
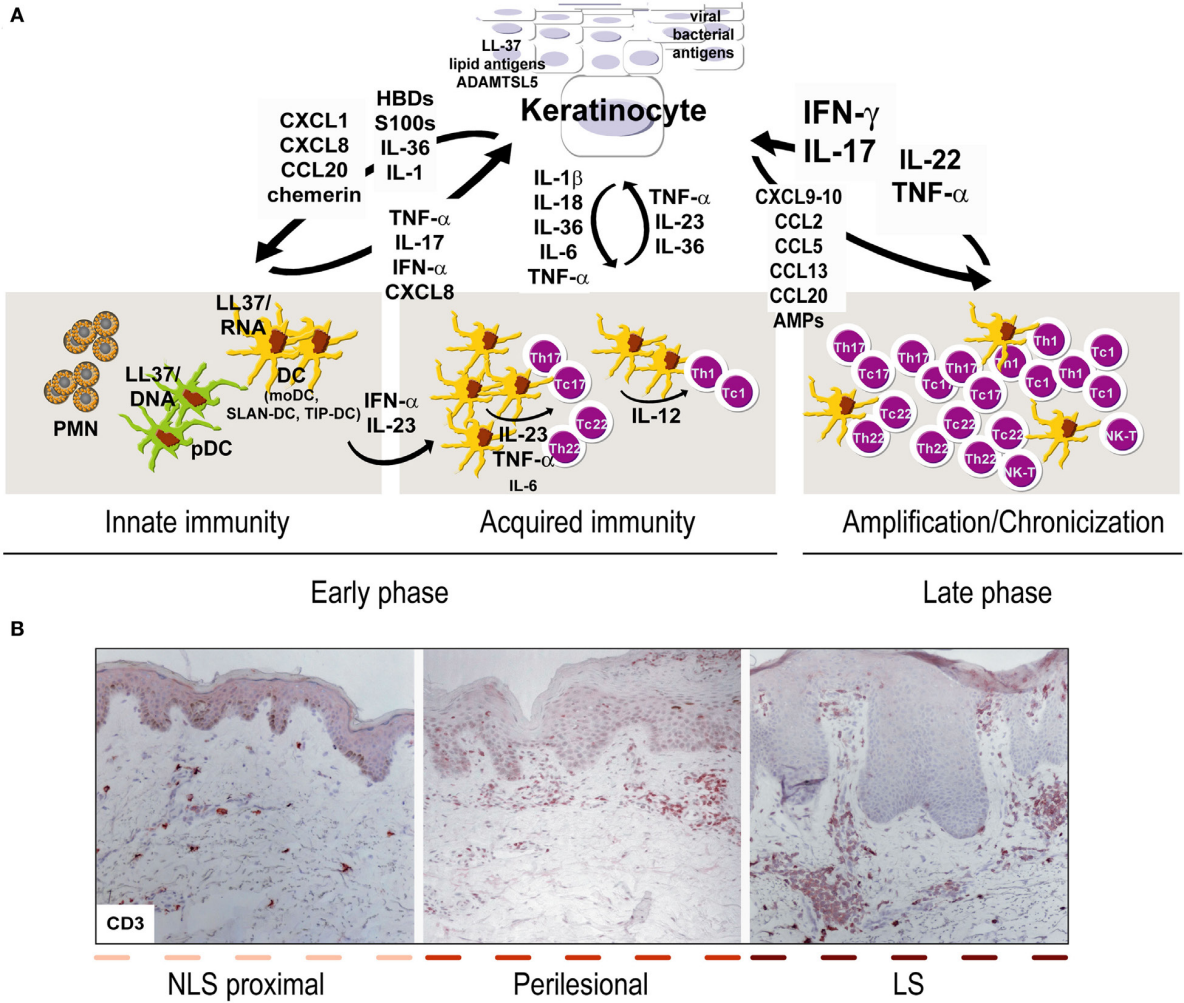
Aberrant interplay of keratinocytes and immune cells in psoriasis. **(A)** Early upstream events in psoriasis include induction of innate immunity pathways and acquired responses, and keratinocytes represent the “key responding” skin cells by producing trigger factors, including LL37/nucleic acid complexes, lipid antigens, and ADAMTSL5, as well as pathogens of viral or bacterial origin. During this initial phase, keratinocytes also produce antimicrobial peptides (AMP), such as β-defensins (HBD) and S100 proteins, together with chemokines and cytokines of IL-1 family. Among AMP, LL37 activates plasmacytoid dendritic cells and myeloid DC (mDC), which routinely patrol uninvolved psoriatic skin, in particular, non-lesional (NLS) skin proximal to lesions, with consequent beginning of the adaptive immune phase. In early phase, SLAN-DC and TIP-DC, highly producing TNF-α, are also present. Hence, DC drive expansion of T lymphocytes, mostly Th17 and Th22 in the beginning and type-1 interferon (IFN)-γ-producing T cells, especially during the chronic phase. During acquired immunity phase, keratinocytes influence DC immune functions by producing cytokines derived from inflammasome pathway, and IL-36. T-cell infiltrate present during late/chronic phase of psoriasis establishes a cytokine milieu, mainly represented by IFN-γ, TNF-α, IL-17, IL-22, which dictates specific and pathogenic gene signatures in keratinocytes, which, thus, overexpress a number of inflammatory mediators. In parallel, under the influence of cytokines, in particular, IL-22 and IL-17, keratinocytes hyperproliferate and show altered differentiative programs. During the amplification/chronicization phase of the disease, unceasing cross-talk between keratinocytes and immune cells further amplifies inflammation and hyperplasia. **(B)** Both early and late phases of psoriasis can be found within the same psoriatic plaque, being it comprehensive of LS, perilesional and adjacent NLS areas, with markers of chronic inflammation (i.e., CD3^+^ T accumulation in the dermis) predominantly present in LS skin [CD3 stainings have been retrieved from Ref. (48)].

Psoriasis is a chronic inflammatory skin disorder involving both innate and adaptive immunity processes. It is caused by the infiltration of distinct effector leukocyte subpopulations in both the epidermis and dermis, which determines hyperproliferation of the epidermis with aberrant differentiation of keratinocytes (1, 2). As a consequence, the epidermis is thickened, with elongated rete ridges forming protrusions into dermis (2, 3). There has been a long debate on pathogenic functions of keratinocytes in psoriasis, and numerous studies have established that hyperproliferation and abnormal differentiation of keratinocytes is a secondary phenomenon induced by immune activation. This “immune” hypothesis, mainly based on dendritic cell (DC) and T cell pathogenic functions, has found confirmation in the efficacy of immune-targeting treatments (4–6). However, psoriasis cannot be considered uniquely as a T-cell-dependent disease, and it is now well known that keratinocytes have a crucial role in triggering the early pathogenic events, as well as in sustaining the chronic phase of the disease (1, 7). Early upstream events occurring in psoriasis include induction of innate immunity pathways and responses, and keratinocytes represent the “first-line responding” skin cells to psoriasis pathogenic environment (8). Upon activation by trigger factors, such as skin trauma and pathogens (i.e., streptococci) or drugs, respectively, keratinocytes become a source of innate immune mediators (Figure 1A). The latter include cationic antimicrobial peptides (AMP), cytokines of IL-1 family, and chemokines active in the recruitment of leukocyte subpopulations of innate immunity, such as plasmacytoid dendritic cells (pDC), neutrophils, mast cells, and macrophages (9, 10). Among AMP, the cathelicidin LL37 has been associated with the development of psoriasis, through its capacity to activate pDC and myeloid DC (mDC), with consequent initiation of the adaptive immune phase (10, 11). DC drive expansion of T lymphocytes, typically T helper (Th)17 and Th22 in the initial phase and interferon (IFN)-γ-producing T cells during the chronic phase of the disease (Figure 1A). T-cell infiltrate present in active psoriatic skin establishes a cytokine milieu that dictates specific gene signatures in keratinocytes, which, thus, overexpress several inflammatory mediators amplifying local immune reactions (12, 13). Unceasing cross-talk between keratinocytes and adaptive immunity cells further intensifies inflammation and may be essential in disease chronicity (Figure 1A). In addition, intrinsic or genetic alterations of keratinocytes in the activation of key signaling pathways induced by immune cell-derived cytokines may be responsible for the typical unbalance between proliferation and differentiation processes, as well as inflammatory signatures observed in psoriatic epidermis (14, 15).

In this review, we will illustrate the multiple functions of keratinocytes during the initiation, maintenance, and amplification of the immune and inflammatory programs associated with psoriasis. A critical role of keratinocytes in triggering and sustaining the innate and adaptive immune responses will be discussed.

## Psoriatic Keratinocytes as Inducers of Innate Immune Responses in the Early Phase of Psoriasis Development

Much efforts have been devoted to understanding the primary pathogenic mechanisms and the cell components responsible for the onset of the disease. Growing evidences propose a fundamental role for keratinocytes. At this initial phase, pDC and neutrophils infiltrate skin plaque lesions, and psoriatic keratinocytes are deeply involved in their recruitment and activation (16). Important studies aimed at identifying the initial trigger of psoriasis demonstrated that injured keratinocytes enable pDC-, concomitantly to mDC-driven immune responses through LL37/nucleic acid complexes, highly released in psoriatic epidermis after skin trauma (11, 17). These multimeric LL37–nucleic acid complexes induce overproduction of type I IFN by pDC as well as TNF-α and IL-6 by mDC (11, 18). A similar mechanism for pDC activation has been described for human β-defensin (HBD)2 and HBD3, other two AMP released by psoriatic keratinocytes (19). Also, macrophage populations, highly present in early lesional skin, can be activated by keratinocyte-derived LL37, which promotes their differentiation toward a proinflammatory signature by recognizing the P2×7 purinergic receptor (20). Remarkably, keratinocyte-derived LL37 also regulates the expression of cytokines of IL-1 family, including IL-36γ, by keratinocytes themselves in paracrine and autocrine loops (10). Furthermore, LL37 induces CXCL8 and CXCL1 chemokines through IL-36R signaling in psoriatic keratinocytes, which would, in turn, determine the recruitment and the burst of neutrophils in lesional skin, typical of the early phase of psoriasis (10). Importantly, LL37 induces CXCL10 and CCL20 in psoriatic keratinocytes and is most likely responsible for the first flare of the acquired immunity established in later phase of psoriasis by DC and Th17 (10).

Other chemotactic factors are expressed by keratinocytes during the induction phase of psoriasis. For instance, the chemokine chemerin, which is also abundantly released by dermal fibroblasts, is responsible for migration and accumulation of BDCA-2^+^ pDC into pre-psoriatic skin, as well as in early psoriatic lesions. Through secretion of IFN-α, pDC activate pathogenic T-cells by inducing the maturation of mDC, and by initiating the mononuclear Th1 responses, which persist in adaptive immunity (21, 22). Chemerin expression by keratinocytes and fibroblasts is lost during later stages of plaque development, and pDC disappear in fully developed psoriasis plaques (16).

Of note, the production of IFN-α by early immigrating pDC favors acquired immune responses also by suppressing IL-1 production by keratinocytes, and, therefore, by shortening skin inflammation (23). In addition, the pathogenicity of IFN-α is also supported by the findings showing its signaling signature in keratinocytes of psoriatic plaques (24), and that psoriasis is exacerbated if patients with psoriasis are treated with IFN-α for unrelated diseases, such as viral infections or tumors, or by imiquimod (IMQ) that induces IFN-α release by pDC *via* toll-like receptor activation (25, 26).

Keratinocyte-derived IL-1α represents an additional inducer of innate immune responses in psoriasis, and it can favor neutrophil and monocyte accumulation during early psoriasis papule formation. IL-1α functions as an alarm signal and is heavily induced in necrotic psoriatic keratinocytes, especially by factors such as cytosolic DNA (27). Concomitantly to IL-1α, self-DNA induces assembling of psoriatic inflammasome containing the DNA sensor AIM2, and, thus, promotes the production and release of IL-1β and IL-18, other two pathogenic member of IL-1 family (27). Interestingly, cathelicidin LL37 can interfere with DNA-sensing inflammasomes, suggesting an antagonistic function for this peptide in the autoinflammatory pathways associated to early psoriatic processes. Critical alterations in the IL-1/IL-1 receptor system have also been found in lesional psoriatic skin (28, 29), with IL-1α and IL-1β being abundant in psoriatic lesions concomitantly to the soluble isoform of the IL-1 receptor antagonist and IL-1-RII decoy receptor. These observations suggest the impairment of negative regulatory mechanisms of IL-1 system in psoriasis. Consistently, transgenic overexpression of IL-1α and IL-1 type I receptor in the skin leads to a pathogenic phenotype in mice resembling psoriasis (30). Other than activating innate immune responses, IL-1α has found to promote keratinocyte and endothelial cell proliferation and activation (31).

## Keratinocyte Involvement in Adaptive Immune Responses in Psoriatic Skin

Psoriasis lesional skin shows many inflammatory CD3^+^ T cells, which progressively accumulate in both the upper dermis and epidermis (1, 2), thus determining the typical epidermal hyperplasia and inflammation picture (Figure 1B). Immunophenotyping of T cells shows that they are mostly activated memory T cells expressing cutaneous lymphocyte antigen (CLA) and belongs to distinct subsets of CD4^+^ and CD8^+^ lymphocytes, Th1/Tc1, Th17/Tc17, and Th22/Tc22 (2). DC activation is determinant in driving T cell responses, even if keratinocytes can trigger and condition DC responses, and, thus, influence acquired immunity in psoriatic skin.

First, keratinocytes have been described as active producers of causative antigens of psoriasis, and the position of dermal mDC at the dermal–epidermal junction, as well as in the epidermis would favor the capture by mDC of keratinocyte-derived extracellular antigens for presentation to T cells, and intracellular antigens *via* cross-presentation. Recently, three autoantigens identified in keratinocytes were found to be involved in the pathogenesis of psoriasis. Among them, LL37 is recognized as autoantigen by circulating CD4^+^ and CD8^+^ T cells with a cytokine and skin-homing receptor profile typical of psoriatic skin T cells (IFN-γ^high^, IL-17^high^, CLA^+^, CCR6^+^, and CCR10^+^), in up to 75% of psoriatic patients (32). Most recently, phospholipase A2 group IVD was identified in psoriatic keratinocytes as important player in the generation of psoriasis autoantigens (33). The latter include non-protein neolipids that are recognized by CD1a-restricted T cells, thereby inducing the production of IL-22 and IL-17A (34). These lipid antigens could be transferred from keratinocytes to neighboring antigen-presenting cells through released exosomes, similarly to what observed for tryptase^+^ mast cells of psoriatic lesions (34). Finally, the disintegrin and metalloprotease domain containing thrombospondin type 1 motif-like 5 (ADAMTSL5), a protein modulating microfibril functions (34, 35) and identified as autoantigen presented by melanocytes in a HLA-C*06:02-restricted fashion (36), has been also recently localized in keratinocytes throughout the psoriatic epidermis (34, 37). Keratinocytes could also activate pathogenic T cells by presenting viral or bacterial products. A relevant presence of human papillomavirus-5 DNA and reactive antibodies against virus-related particles have been found in psoriasis (38). Infections by *Streptococcus* commonly associate with psoriasis, and streptococcus-derived superantigens can be presented to T lymphocytes by MHC class II-bearing keratinocytes. Psoriatic antigens are also supposed to be keratinocyte-derived molecules sharing structural homology with streptococcal proteins, which could, therefore, induce autoreactive T-cell responses against skin components (39).

Second, DC function and maturation in the psoriatic context is also depending on mediators released by keratinocytes during the innate immunity phase. Among them, IL-36 cytokines, a subgroup of IL-1 family, comprising the IL-36α, β, and γ agonists, strongly upregulated in psoriatic lesional epidermis, were found to influence DC function (40, 41). In humans, IL-36 cytokines activate mDC by increasing the proportion of cells with a strong CD83, CD86, and HLA-DR membrane expression and induce the secretion of IL-1β and IL-6, thus promoting the Th17 differentiation (42). Consistently, keratinocyte/DC cross talk mediated by IL-36 was essential in driving IMQ-induced psoriasiform dermatitis in mice. In this model, IL-36 signaling controlled the aberrant IL-23/IL-17/IL-22 axis and disease development (43). Similarly, IL-18 highly released by keratinocytes downstream to inflammasome pathway is involved in the recruitment of IL-18R-bearing DC to inflammatory sites characterized by Th1 responses, as in psoriasis. IL-18 from keratinocytes in synergy with IL-12 plays a role in the Th1 response, primarily by inducing IFN-γ in psoriatic lesions (44). In addition, recent evidence suggests that γδ T cells infiltrating can produce IL-17 *via* IL-23 in presence of IL-18 (45). Together with IL-18, mature IL-1β is also produced by keratinocytes as result of inflammasome activation, and influence DC-mediated immune responses. A number of evidence links IL-1β and Th17 pathways in psoriasis pathogenesis both in mice and in humans. Transgenic overexpression of IL-1β is responsible for massive presence of Th17 cells in the skin, as well as for the inflammatory psoriasisform phenotype of mice (46). Furthermore, the IL-23-dependent differentiation of human Th17 cells relies on the copresence of IL-1β (27).

## Keratinocytes are a Reservoir of Inflammatory Mediators, Which Sustain Persistence of Psoriasis Lesions

During the chronic phase of psoriasis, expansion and activation of T and DC subpopulations in lesional areas establishes a definite cytokine micromilieu, mostly represented by IFN-γ, IL-17, TNF-α, and IL-22. Keratinocytes bear receptors for these cytokines and potently respond by further releasing cytokines. Under the effects of these cytokines, keratinocytes also show altered proliferative and differentiation programs, as well as enhanced resistance to apoptosis (1, 47–49).

Each cytokine regulates distinct responses in keratinocytes with a certain degree of overlap in gene expression induction/inhibition. Transcriptional profiling studies conducted on lesional psoriatic skin showed that the IFN-γ-signature predominates, with the upregulated expression of approximately 400 genes dependent on signal transducer and activator of transcription 1 (STAT1), the preferential IFN-γ molecular node (50). *In vitro*, IFN-γ represents the most potent stimulus for keratinocyte inflammatory and immune activation, as it regulates the expression of approximately 1,200 genes (51). Importantly, the IFN-γ capability to induce inflammation in psoriasis was demonstrated by a study showing that IFN-γ injection in clinically uninvolved psoriatic skin determines a transcriptomic profile and inflammatory cell infiltration similar to lesional skin (51). Indeed, the same results were obtained with healthy volunteers, indicating that other cytokines specifically contribute to the psoriatic phenotype (51). IFN-γ induces in psoriatic keratinocytes key-disease mediators, such as chemokines attracting monocytes and Th1 and Th17 cells (CCL2, CCL5, CXCR3 ligands), DC (CCL13, CCL20), or CCR10^+^ skin-homing CLA^+^ T cells highly infiltrating psoriatic skin (1). HBDs and S100 proteins are also potently induced by IFN-γ in keratinocytes, alone or in synergy with TNF-α. Most of the IFN-γ-induced effects in keratinocytes are potentiated by TNF-α, which signals mainly by activating NF-κB, a transcription factor regulating gene expression frequently in collaboration with STAT1. TNF-α induces expression of ICAM-1 on keratinocytes, permitting the adhesion of circulating leukocytes. Moreover, TNF-α stimulates keratinocyte production of several chemokines active on neutrophils, T cells, and DC (CXCL1, CXCL2, CXCL8, CCL2, CCL5), as well as pro-inflammatory cytokines, such as IL-6 and IL-1, which help maintain Th17 cells (1, 2). Importantly, TNF-α induces IL-36γ in psoriasis lesions, which in turn promote expression of AMP and chemokines recruiting neutrophils and Th17 cells, as well as interfere with terminal differentiation and cornification process of psoriatic epidermis (52). The increased production of IL-36γ is also associated with the presence of Th17 lymphokines in psoriatic skin lesions, as IL-17 and IL-22 strongly induce its expression (52, 53).

Keratinocytes are also strongly influenced by IL-17 and upregulate chemokines (CXCL1, CXCL3, CXCL5, CXCL8, CCL20), AMPs (LCN2, HBD-2, S100A7), and immunomodulatory molecules, such as ICAM1, in response to this cytokine. In addition, IL-17 stimulates LL37 autoantigen production (54–56) and delay terminal differentiation of keratinocytes. IL-17 activates NF-κB, possibly through the IKBζ transcriptional regulator, as well as Act-1 intracellular pathway, which is required for IL-17 induction of keratinocyte host defense genes and inhibition of differentiative programs in keratinocytes (57). IL-17 also induces IL-19, a member of IL-20 family, which has mitogenic effects on keratinocytes themselves (58).

Th17- or Th22-derived IL-22 cytokine also acts pathogenically on psoriatic keratinocytes by promoting release of chemokines (CXCL1, CXCL2, CXCL8, CCL20), AMPs (HBD-2, HBD-3, S100 proteins), as well as inducing their proliferation and de-differentiation (59, 60). Binding of IL-22 to its receptor, the expression of which in the skin is confined to keratinocytes, mediates epidermal acanthosis through the activation of STAT3. These observations may explain the increased STAT3 expression in the epidermal compartment and the pathogenicity of STAT3 overexpression in the epidermis of transgenic mice (61). Although studies demonstrated a central role of IL-22 in psoriasis pathogenesis, this cytokine induces a limited panel of genes compared to IL-17, as detected in human lesional psoriatic skin (55), and antibodies neutralizing IL-22 failed to show a therapeutic effect in patients with psoriasis.

Collectively, IFN-γ, IL-17, TNF-α, and IL-22 can cause keratinocyte production of chemokines, cytokines, and AMPs, as well as concur to derange proliferative and differentiative programs of the epidermis. This becomes a self-amplifying loop, where these products and altered homeostasis act back on T cells, DC, and neutrophils to perpetuate the cutaneous inflammatory processes.

## Conclusion

Currently, there are no meaningful hypotheses to explain certain typical features of psoriasis including the sharply demarcated presentation of the lesions and the more frequent localization of the lesions in certain anatomical sites (i.e., scalp, extensor body areas), although a skin/keratinocyte-specific mechanism may be involved. In contrast, the fluctuant behavior of psoriasis with phases of remission and recrudescence may be related to prevailing regulatory and effector immune mechanisms. Activated T lymphocytes are required for the development and persistence of immune responses in psoriatic skin, even though psoriasis cannot be considered uniquely as a T cell-dependent disease. In fact, the presence of type-17, type-1, and type-22 subtypes in inflamed skin could be the result of a common response to antigens, also effective in subjects not predisposed to psoriasis. Indeed, this paradox could be explained either by the presence of other not yet defined psoriasis-related cytokines in skin lesions, or by the intrinsic aberrant response of psoriatic keratinocytes to cytokines or other effector molecules. Several studies showed that genetic defects in keratinocytes are fundamental for psoriasis development. An increasing number of specific single-nucleotide polymorphisms (SNP), found in genes controlling T-cell commitment and keratinocyte inflammatory activation as well as proliferation and differentiation processes in the epidermis, has been associated with psoriasis (14). Among them, a number of SNPs were found in genes encoding molecules involved in IL-17 or TNF-α responses, even though functional studies correlating their presence to keratinocyte susceptibility to these cytokines are lacking and controversial. For instance, allelic variants were found in *NFKBIZ, TRAF3IP2*, and *TNFAIP3* genes (62) encoding IKBζ, Act-1 and the Act1-dependent A20 protein, respectively, all involved in IL-17 molecular signaling (57). However, no clear evidence linking these SNPs to enhanced or reduced responses of keratinocytes to IL-17 exist. In fact, Act-1 gene variants overexpressed in human keratinocytes could decrease as well as enhance Act-1-mediated IL-17 signaling, depending on the SNP type. Variants of *NFKBIZ* could also influence keratinocyte response to TNF-α since IKBζ is a transcriptional cofactor of p50 subunit of NF-κB, the main mediator of TNF-α signaling. In addition, NF-κB activity is regulated by CARD14, a protein of “signalosome” complex involved in activation of innate immunity molecules (i.e., IL-36γ, CXCL8, and CCL20), whose gene shows different allelic variants associated to psoriasis (63). Although a recent study clarified the impact of a CARD14 missense variant (Card14ΔE138), whose expression in mice determined a severe psoriatic phenotype (64), the effects of CARD14 SNPs on TNF-α- or IL-17-induced responses in keratinocytes remain to be defined.

Future studies must consider these genetic aspects, especially those concerning the relationship between genetic determinants and keratinocyte inflammatory responses to psoriasis-related cytokines. Moreover, genetic data need to be further integrated with analyses of the cytokine milieu specifically characterizing the psoriatic patient. This will permit to predict the responsiveness of psoriatic patient to a specific therapy, thus implementing a personalized medicine approach.

## Author Contributions

Each author has contributed in the ideation and writing of the manuscript, and each author has checked the final version of the paper.

## Conflict of Interest Statement

The authors declare that the research was conducted in the absence of any commercial or financial relationships that could be construed as a potential conflict of interest.
